# Supplementary material for: Cone Photoreceptor Cell Segmentation and Diameter Measurement on Adaptive Optics Images Using Circularly Constrained Active Contour Model
Source: Invest Ophthalmol Vis Sci. 2018 Sep;59(11):4639–52. doi: 10.1167/iovs.18-24734 (PMC6154284; doi:10.1167/iovs.18-24734)
Supplement: Supplement 1 [file iovs-59-10-59_s01.pdf]

Supplemental Figures and Tables

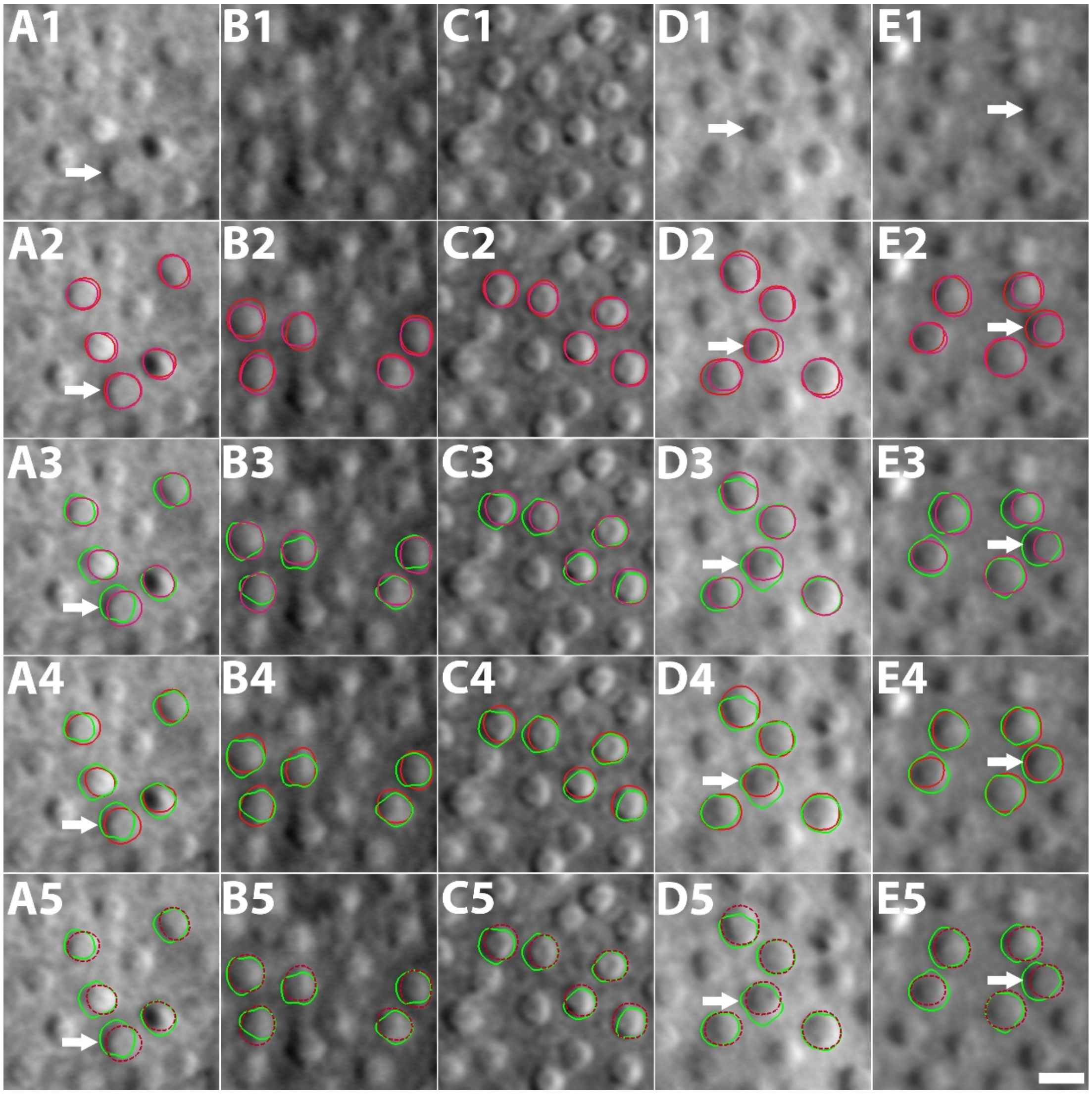

Supplemental Figure 1. Repeatability study of cone segmentation results for subjects 1, 4, 5, 7 and 9, corresponding to each column (A to E). Green contours, automated segmentation; red contours, manual marking. (Row 1) Input images. (Row 2) Manual segmentation on the same cone photoreceptors at two different timepoints, 8.5 months apart. (Row 3) Comparison of CCACM cone segmentation with the initial manual segmentation. (Row 4) Comparison of CCACM cone segmentation with the second manual segmentation. (Row 5) Comparison of CCACM cone segmentation with the averaged manual segmentation (red dash contours). A cone indicated by white arrows in (A1-A5) shows that the grader has a consistent criterion over 8.5 months even though the criterion is slightly different from that of CCACM. Although most of cones were consistently segmented after 8.5 months, some cones, such as the one in (E1-E5), shows significant variation. Despite a few cones being over-segmented by CCACM (D1-D5), there is good overall agreement between CCACM and manual segmentation. Scale bar, 10 μm.

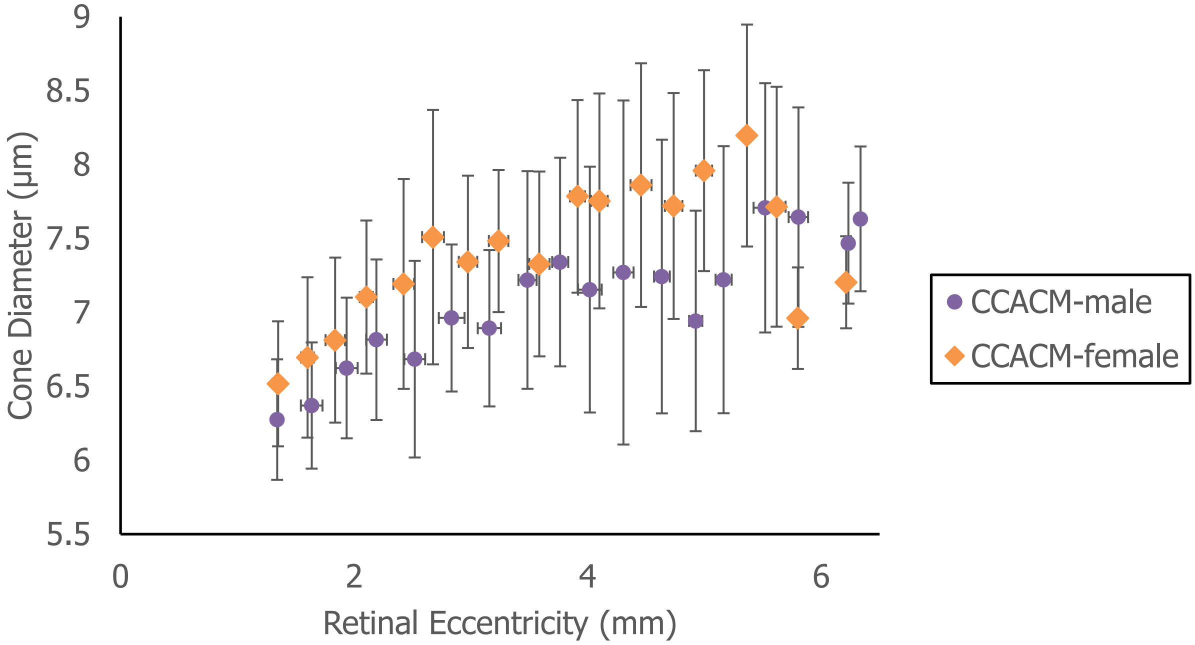

Supplemental Figure 2. Comparison of cone diameters calculated from circularly-constrained active contour model in five male and five female subjects Vertical bars denote the one standard deviation of cone diameters, and horizontal bars standard deviation of eccentricities.

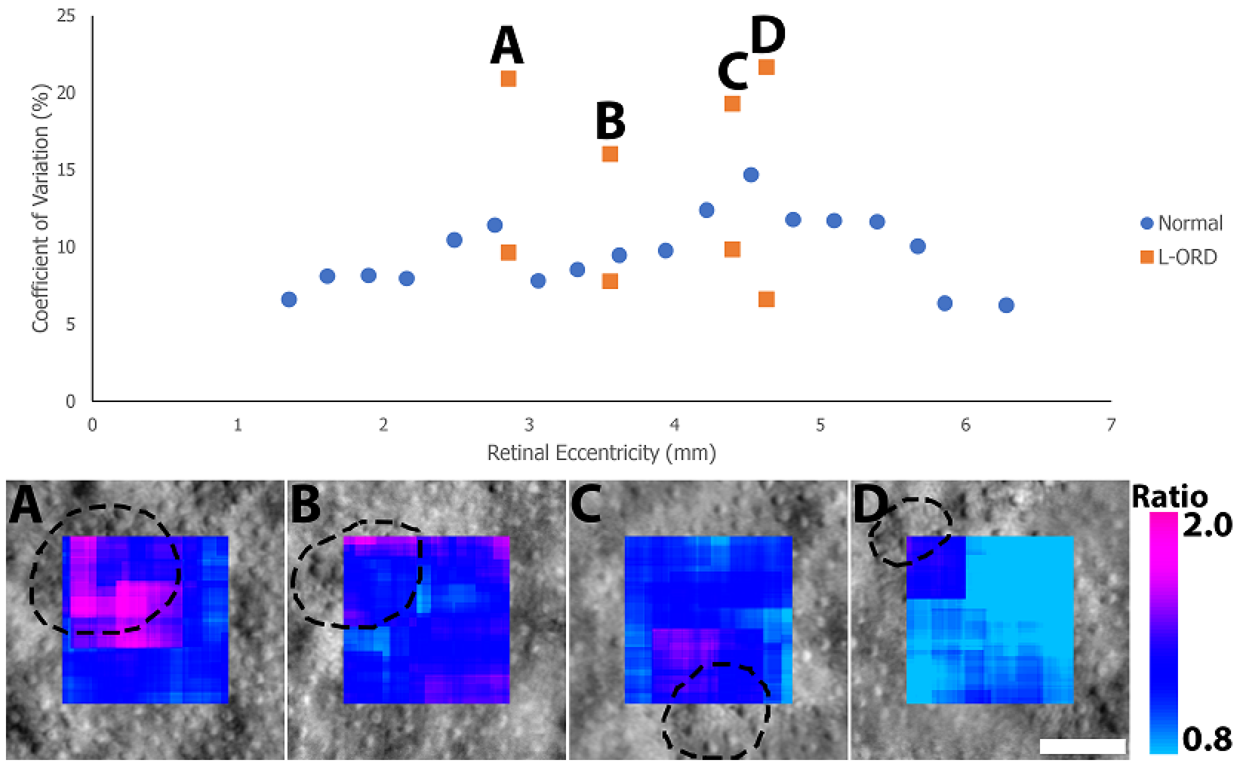

Supplemental Figure 3. Coefficient of variation of cone diameters at different eccentricity in healthy subjects along temporal direction, where coefficient variation is measured through cone diameter standard deviation over its mean (top row, blue dots). Minimum and maximum coefficient of variation values across four ROIs from a L-ORD patient (**Figure 7**) are also plotted (top row, brown squares). In each ROI, a sliding window was used across the image to calculate the coefficient of variation centered at various points across the ROI. The ratio of coefficient of variation values of the L-ORD patient relative to the expected value from healthy subjects at the appropriate eccentricities are depicted with the color maps (bottom row). Larger coefficient of variation values tend to occur near lesions. Scale bar, 50μm.

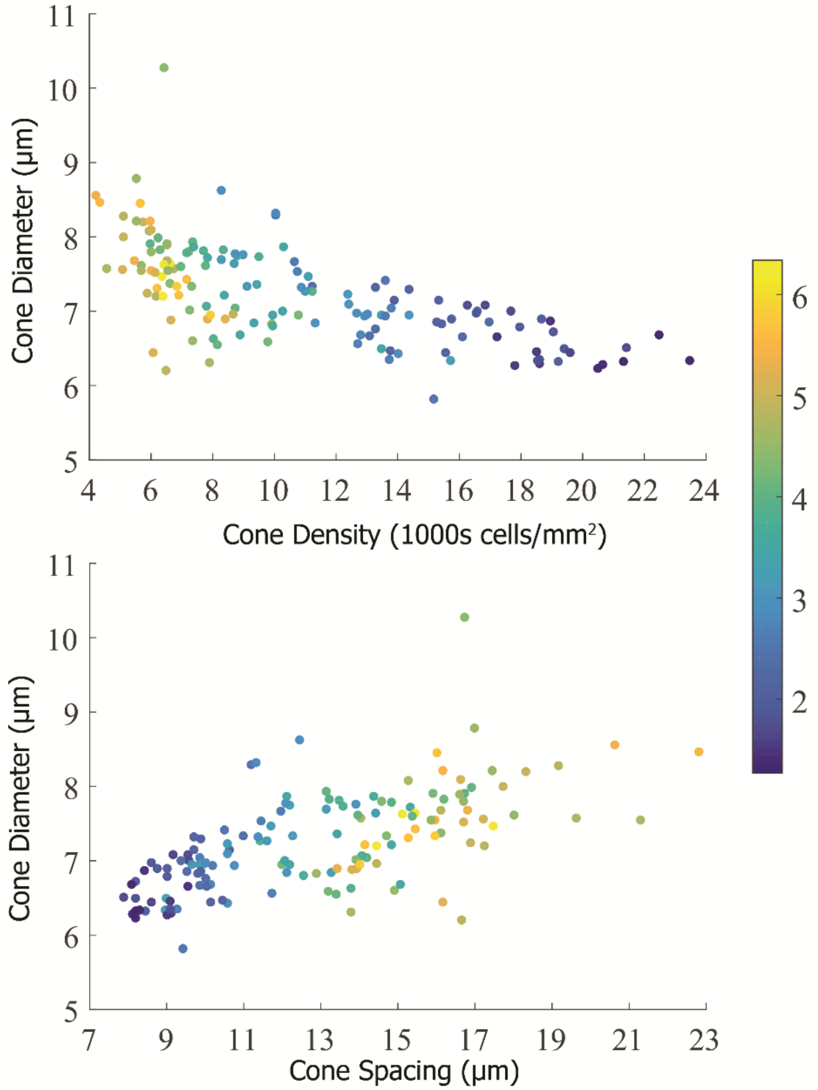

Supplemental Figure 4 – Correlations between cone density and spacing with cone diameter measured in the same cells (see **Figure 6**). Each dot represents data from one ROI, color-coded for eccentricity (color map on right, in mm). Cone diameters represent the average diameters across the ROI.

Supplemental Table 1. Subject List

Subject	Eye	Age	Sex	Refraction(D)	Axial Length (mm)
1	OS	30	M	plano	23.53
2	OD	22	F	plano	23.77
3	OS	24	M	-0.75,+0.5×92	23.85
4	OD	40	M	-5.5,+1×92	26.20
5	OD	20	M	plano	22.96
6	OS	23	F	-1.0,+0.0	24.74
7	OS	29	F	plano	23.38
8	OS	29	F	plano	23.42
9	OD	24	M	-5.75,+2.0×104	26.57
10	OD	22	F	-0.5,+0.5×176	23.82
11*	OD	55	M	-1.5,+0.25×135	23.26

* This subject is the patient with late-onset retinal degeneration.

Supplemental Table 2. Segmentation accuracy on ten subjects

Subject	ASD (μm)	RMSD (μm)	MSD (μm)	ADD (μm)	RDD (%)
1	0.58	0.71	1.48	0.62±0.49	7±5
2	0.72	0.83	1.59	0.64±0.55	6±5
3	0.54	0.64	1.39	0.65±0.52	9±5
4	0.63	0.76	1.53	0.71±0.65	7±6
5	0.63	0.76	1.49	0.81±0.59	9±7
6	0.53	0.65	1.38	0.78±0.53	10±7
7	0.61	0.74	1.51	0.64±0.30	7±4
8	0.55	0.67	1.39	0.56±0.37	6±5
9	0.82	0.98	1.85	0.96±0.51	8±3
10	0.68	0.80	1.62	0.72±0.63	8±5

ASD: average symmetric contour distance.
RMSD: root mean square symmetric contour distance.
MSD: maximum symmetric contour distance.
ADD: absolute diameter difference.
RDD: relative diameter difference.

Supplemental Table 3. Repeatability of cone contour marking from the same grader.

Method	ASD (μm)	RMSD (μm)	MSD (μm)	ADD (μm)	RDD (%)
First VS. Second	0.42±0.10	0.51±0.12	1.02±0.21	0.50±0.36	6±5
First VS. Segmentation	0.62±0.12	0.74±0.14	1.48±0.23	0.70±0.50	9±7
Second VS. Segmentation	0.58±0.09	0.70±0.10	1.40±0.18	0.65±0.52	9±7
Average VS. Segmentation	0.57±0.08	0.68±0.09	1.36±0.18	0.62±0.48	8±6

Five cones from each of the ten split detection AOSLO images from ten subjects were repeatedly marked by the same grader with 8.5 months apart. The repeatability of segmentation accuracy was evaluated by comparing automated segmentation results with the first time manual marking, segmentation results with the second time manual marking, segmentation results with the average contours from two manual marking, as well as comparison between the first time manual marking and the second time manual marking.

ASD: average symmetric contour distance.
RMSD: root mean square symmetric contour distance.
MSD: maximum symmetric contour distance.
ADD: absolute diameter difference.
RDD: relative diameter difference.
